# Effects and Components of Herb Pair Huanglian-Banxia on Diabetic Gastroparesis by Network Pharmacology

**DOI:** 10.1155/2021/8257937

**Published:** 2021-10-18

**Authors:** Guoqiang Liang, Lurong Zhang, Guorong Jiang, Xuanyi Chen, Yang Zong, Fei Wang

**Affiliations:** ^1^Central Laboratory, Suzhou TCM Hospital Affiliated to Nanjing University of Chinese Medicine, Suzhou, 215000 Jiangsu, China; ^2^Clinical Pharmaceutical Laboratory of Traditional Chinese Medicine, Suzhou Academy of Wumen Chinese Medicine, Suzhou, 215000 Jiangsu, China; ^3^Department of Gynecology, Suzhou TCM Hospital Affiliated to Nanjing University of Chinese Medicine, Suzhou, 215000 Jiangsu, China

## Abstract

Diabetic gastroparesis (DGP) is a serious and chronic complication of long-standing diabetes mellitus, which brings a heavy burden to individuals and society. Traditional Chinese medicine (TCM) is considered a complementary and alternative therapy for DGP patients. Huanglian (Coptidis Rhizoma, HL) and Banxia (Pinelliae Rhizoma, BX) combined as herb pair have been frequently used in TCM prescriptions, which can effectively treat DGP in China. In this article, a practical application of TCM network pharmacological approach was used for the research on herb pair HL-BX in the treatment of DGP. Firstly, twenty-seven potential active components of HL-BX were screened from the TCMSP database, and their potential targets were also retrieved. Then, the compound-target network and PPI network were constructed from predicted common targets, and several key targets were found based on the degree of the network. Next, GO and KEGG enrichment analyses were conducted to obtain several significantly enriched terms. Finally, the experimental verification was made. The results demonstrated that network pharmacological approach was a powerful means for identifying bioactive ingredients and mechanisms of action for TCM. Network pharmacology provided an effective strategy for TCM modern research.

## 1. Introduction

Diabetic gastroparesis (DGP) is a serious and chronic complication of diabetes characterized with the disorder of gastric motility and delayed gastric emptying in the absence of mechanical obstruction [1]. A recent and prospective study, called DCCT-EDIC, found that the morbidity of DGP was about 47% in diabetic patient with a prolonged history [2]. The treatment of DGP is aimed at controlling high-level blood glucose and improving gastrointestinal function. It was reported that some chemical drugs, such as domperidone and metoclopramide, improved some symptoms of gastroparesis. However, these therapeutic drugs have shown limited efficacy but fail to stop or reverse disease progression. Moreover, adverse effects further restricted the clinical treatment of DGP [1, 3–5]. Therefore, as a complicated disease [6], DGP may require complex therapeutic approaches such as traditional Chinese medicine (TCM).

In TCM, DGP is treated as “Pi man” and “Wei Pi” and so on. Its clinical symptoms include nausea, vomiting, belching, sense of fullness, early satiety, bloating, postprandial fullness, and abdominal discomfort [7]. It is considered that the main cause of DGP is the imbalance of *Qi* in ascending and descending. One of therapeutic principle named “Xin Kai Ku Jiang” has long-term clinical practice in the prevention and treatment of DGP. There were some TCM prescriptions recorded in *Treatise on Cold Damage and Miscellaneous Disease*, such as Ban-Xia-Xie-Xin decoction and Huang-Lian-Ban-Xia-Jie-Du decoction, which were used to improve gastrointestinal function [8]. As the simplest form and the concentrated representative of this prescription, herb pair Huanglian-Banxia (HL-BX) essentially reflected the basic idea of those prescriptions in treatment of DGP [9]. HL-BX has the function of regulating the gastrointestinal tract and *Qi* and balancing *Yin* and *Yang*. It consists of two herbs: Huanglian (HL) and Banxia (BX). The properties of BX are spicy and warm, which can dry dampness and phlegm in the spleen and descend rebellious stomach *Qi* in order to harmonize the middle energizer. And HL has the features of bitter and cold, which plays roles in draining dampness and clearing heat, and also can erase heat concentration in *Qifen* of the middle energizer. It has been reported that HL had a significant hypoglycemic effect which made its early application in the treatment of diabetes and its complications [10]. The aqueous extract of HL inhibited the gastric emptying induced by dopamine [11]. Baicalein from BX effectively prevented the 6-OHDA-induced dopaminergic dysfunction through an antioxidative action [12]. *β*-Sitosterol from BX decreased the levels of plasma glucose and increased the levels of insulin and hemoglobin and protein expression of PPAR*γ* and GLUT4 [13]. Although many researches have indicated that BX-HL is composed of multiple biologically active compounds and had multimechanisms, the exact ingredients and mechanisms of BX-HL remain unknown due to its complex nature, as well as a lack in appropriate methods.

TCM network pharmacology, based on the same integrity and systematic characteristics as TCM theory, was used to predict potential effective ingredients, targets, and the mechanism of TCM from a systematic perspective and at the molecular level [8, 14, 15]. It has been widely used in the prediction of TCM and its prescriptions on diabetes and its complications. For example, the potential antidiabetic ingredient from the traditional Ge-Gen-Qin-Lian decoction formula, 4-hydroxymephenytoin, was successfully found and validated by network pharmacology [16]. The mechanism of action for Buxuhuayu decoction in diabetic treatment was proved to promote diabetic ulcer healing by activating the PI3K/Akt/eNOS signalling pathway based on network pharmacology [17]. The role of Cordyceps in the treatment of diabetic nephropathy was elucidated by network pharmacology to be comprehensive *via* multiple pathways [18]. The molecular mechanisms of Ban-Xia-Xie-Xin decoction in treating DGP were affirmed to be a complex network with multitarget and multipathway by network pharmacology [19]. Hence, to better understand the effective components and the molecular mechanisms of the therapeutic effects of HL-BX on DGP, utilization of network pharmacology technology to seek the components and potential pathways of HL-BX in the treatment of DGP is completely consistent with the concept of “Disease-Gene-Target-Medicine” and the holistic view of TCM. Therefore, it is promising to clarify the characteristics of multi-ingredient, multipathway, and multitarget of HL-BX in the treatment of DGP by this method.

**Figure 1:**
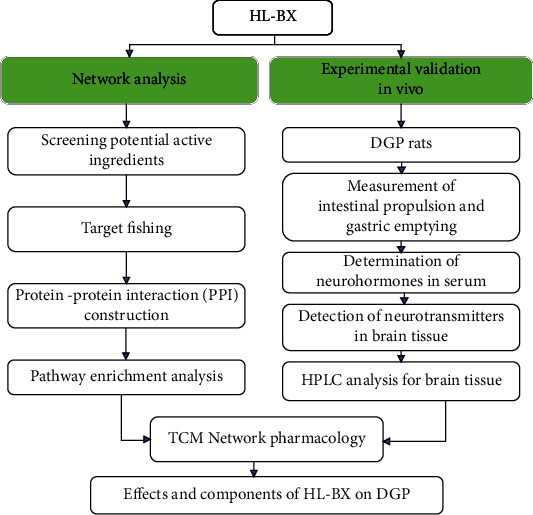
The flow chart of HL-BX research on DGP.

In the present study, the active ingredients, the potential targets, and pathways in HL-BX were investigated based on TCM network pharmacology analysis and the anti-DGP activities were experimentally validated *in vivo*. Firstly, the active ingredients and the potential targets of HL-BX were screening potential active ingredients from multiple databases. Then, the compound-target network and PPI network were constructed from predicted common targets, and several key targets were found based on the degree of the network. Next, GO and KEGG enrichment analyses were conducted to obtain several significantly enriched terms. Finally, the experimental verification *in vivo* was carried out. The levels of neurotransmitters and neurohormones were observed, and the components absorbed into the brain were detected. The flow chart is shown in Figure 1.

## 2. Materials and Methods

### 2.1. Materials and Chemicals

Standards of berberine (Batch No. 20180707, purity > 95%) were gotten from Shanghai Pure One Bio Tech Co., Ltd. (Shanghai, China). Standards of dopamine (DA) and norepinephrine (NE) (Batch No. N-5785, H 8502-5G, respectively; purity > 95%) were gotten from Sigma (St. Louis, MO). The activated carbon (Batch No. 07093005) was purchased from Wuxi Zhanwang Chemical Reagent Co., Ltd. (Wuxi, China). HPLC grade methanol and acetonitrile were purchased from Scharlab, S. L. (Barcelona, Spain). The water for HPLC was purified by an ultrapure water instrument. Other reagents used in the present study were of analytical grade.

### 2.2. Animals

Male Sprague-Dawley rats (8 weeks, approximately 200 g each) were purchased from the animal experiment centre of Zhaoyan Co., Ltd. (Suzhou, China; animal certificate no. SCXK (SU) 2018-0006). The experiments were performed in accordance with protocols approved by the institutional ethic committee, and this study also complied with the criteria in Guide for the Care and Use of Laboratory Animals of Suzhou Hospital of Traditional Chinese Medicine (Permit Number: SCXK(SU) 2019.0012). All rats were housed at 23 ± 2°C and 60 ± 10% humidity using a 12/12 h light/dark cycle in the Experimental Animal Centre of Suzhou Hospital affiliated to Nanjing University of Chinese Medicine. The rats were allowed to acclimatize for 1 week before the experiment. All animals were allowed access to water ad libitum and standard laboratory chows. The chows consisted of either a normal diet or a high-sucrose/high-fat diet. The normal diet was provided by the Experimental Animal Centre of Suzhou Hospital affiliated to Nanjing University of Chinese Medicine. The high-sucrose/high-fat diet was composed of five ingredients: normal diet, cooked lard, sucrose, milk powder, and eggs (58 : 15 : 20 : 5 : 2 weight ratio) [20].

### 2.3. Preparation of HL-BX Extract

HL-BX is composed of two herbs: Coptidis Rhizoma, the tuber of *C. chinensis* (Huanglian, HL), and Pinelliae Rhizoma, the rhizome of *P. ternata* (Banxia, BX). The herbs were both purchased from Suzhou Chunhuitang Co., Ltd. (Suzhou, China). The identification of herbs was according to the standards of Coptidis Rhizoma and Pinelliae Rhizoma in *Pharmacopoeia of the People's Republic of China* (Part 1, 2020 Edition, Chinese Medical Science and Technology Press) by Dr. Guorong Jiang. HL-BX powder was prepared as follows: according to Ban-Xia-Xie-Xin decoction recorded in *Treatise on Cold Damage and Miscellaneous Disease*, the weight ratio of HL and BX was 1 : 4. HL-BX was soaked in 3 times amount of distilled water for 0.5 h; then, the solution was refluxed for 1.5 h with 8 times amount of water, and the filtrate was collected by filtration. The drug residue was further mixed with 6 times amount of water and refluxed for 1 h. The filtrate was combined, concentrated in an appropriate amount, frozen at -20°C overnight, and lyophilized to powder. The weighted result was noted: 1000 g crude herbs got 237.7 g powder, so the yield was 23.77%. For the experimental requirements, 0.43, 1.35, and 4.3 g crude herbs/kg (abbreviation: 0.43, 1.35, and 4.3 g/kg) HL-BX powder solution was made by weighing a certain amount of HL-BX powder in distilled water according to the yield of the drug.

### 2.4. Network Pharmacology-Based Prediction of HL-BX on DGP

#### 2.4.1. Database Building of Chemical Ingredients

The Traditional Chinese Medicine System Pharmacology (TCMSP) [21] database (http://tcmspw.com/tcmsp.php, update in 2014-5-31) was used to predict the active ingredients of HL and BX. The principle for screening potential active ingredients was defined as follows: oral bioavailability (OB) ≥ 30% and drug − likeness (DL) ≥ 0.18 [22].

#### 2.4.2. Database Building of Compound Targets and Disease Targets

To predict the corresponding targets of the active ingredients of HL-BX, the corresponding molecule structure information was found in PubChem [23] (https://pubchem.ncbi.nlm.nih.gov/) through the TCMSP database. The targets were then fished and collated with a Swiss Target Prediction [24] web server (http://www.swisstargetprediction.ch/) using a similar set approach. Multiple databases were used as the sources to collect and organize DGP-related targets. The databases included GeneCards [25] (https://www.genecards.org/, version 4.14), DrugBank [26] (https://www.drugbank.ca/, update 2020-4-20), Online Mendelian Inheritance in Man [27] (OMIM; https://omim.org/, update 2020-4-20), and Therapeutic Target Database [28] (TTD; http://db.idrblab.net/ttd/, update 2019-11-11). “Diabetic gastroparesis” was used as the keyword, and then, the DGP targets were further sorted.

#### 2.4.3. Network Construction and Mechanism Analysis

The predicted targets of the active ingredients of HL-BX were matched with the DGP-related targets. The “draw Venn diagram” (http://bioinformatics.psb.ugent.be/webtools/Venn/) was used to identify overlapping targets as potential key targets for HL-BX in the treatment of DGP. Next, the abovementioned overlapping therapeutic targets were subjected to Protein-protein Interaction (PPI) analysis by using the STRING database [29] (https://string-db.org/, version 11.0). Parameters used for species and confidences were “Homo sapiens” and “medium confidence (0.400)”, respectively. All other parameters were at default settings. The data were saved in a Tab Separated Value (TSV) format. Then, Database Visualization and Integrated Discovery system (DAVID, https://david.ncifcrf.gov/home.jsp, version DAVID 6.8) and Kyoto Encyclopedia of Genes and Genomes (KEGG, http://www.genome.jp/kegg/) were used to enrich the analysis for the characterization of target-pathway interactions. In the network, the size of the nodes represented the size of the degree. All the networks were visualized by Cytoscape 3.7.2 [30] (https://cytoscape.org/). In this study, the interrelationships among constituents, potential targets, and inherent pathways were further hunted for investigating the actions of HL-BX on DGP.

### 2.5. Effects of HL-BX on DGP In Vivo

#### 2.5.1. Animal Model

After overnight fasting, blood glucose level (<6.1 mmol/L) was determined from the tail vein by using an Accu-Chek® Advantage blood glucose meter and test strip (Roche, Indianapolis, IN, USA). The rats were induced by a single intraperitoneal injection of streptozotocin (STZ, Sigma-Aldrich, St. Louis, MO, USA) at 60 mg/kg, prepared in 0.1 M citrate buffer. Rats in the control group were injected with equal volume of citrate buffer. Three days later, fasting blood glucose (FBG) was determined from the tail vein. The rats with a level of 11.1~30.0 mmol/L were considered a diabetes model (among them, 3 rats died after STZ injection), which were subsequently fed with a high-sucrose/high-fat diet for 4 weeks. Then, the DM rats with abdominal swelling and weight loss were considered a DGP model [20, 31, 32]. The DGP rats were randomly distributed into four groups (ten rats in each group). Rats in HL-BX groups were treated with 0.43, 1.35, and 4.3 g/kg/day (1×, 3×, and 10× of clinical dose in adults, respectively) *via* intragastric administration. Saline solution was given to the rats in the model group. All rats were fed with a normal diet and free access to drinking water during the administration, while ten rats as the control group were only fed with normal diet all the time. After intragastric administration for 3 weeks, all rats were anesthetized and sacrificed through cardiac puncture. The serum and brain tissue were collected for additional observations.

#### 2.5.2. Measurement of Gastric Emptying and Intestinal Propulsion

After three weeks of treatment, the changes in gastric emptying and intestinal propulsion were evaluated as described previously [33]. The rats were fasted for 18 h prior to the experiment and given a suspension of 10% (*w*/*v*) activated charcoal in aqueous Arabic gum (5% *w*/*v*) (*W*) by gavage. After the suspension was absorbed for 30 minutes, the rats were sacrificed. After exposure by laparotomy, the stomach and small intestine were carefully removed to observe the leading edge of the activated charcoal in the intestine. The stomach was weighed (W1) and cut. The stomach was cleaned off the rest of activated charcoal by immersing in 0.9% saline solution. After dry-blotting with absorbent paper to remove any surface moisture, the stomach was weighed again (W2). The length of the small intestine from the pylorus to the ileocecal junction (L1) and the length of charcoal powder (L2) were measured. The gastric emptying rate and intestinal propulsion rate of the rats were calculated according to the following formula:

(1) $$\begin{array}{c} \text{Gastric emptying rate }\left(\%\right)=\left(\frac{1‐\left(\text{W}1‐\text{W}2\right)}{W}\right)*100\%,\\ \text{Intestinal propulsion rate }\left(\%\right)=\frac{\left(\text{L}1‐\text{L}2\right)}{\text{L}1}*100\%. \end{array}$$

#### 2.5.3. ELISA Assays of Neurohormones

The concentrations of motilin (MLT), somatostatin (SS), vasoactive intestinal peptide (VIP), substance P (SP), glucagon-like peptide-1 (GLP-1), and 5-hydroxytryptamine (5-HT) in the serum were quantitatively measured by using the commercially available ELISA kits (Shanghai Banyi Biological Technology, Shanghai, China) according to the manufacturer's protocols.

#### 2.5.4. HPLC Analysis for Neurotransmitters and Components in Brain Tissue

*(1) Preparation of Brain Tissue Homogenate*. Brain tissue was homogenized on ice with 0.8 M perchloric acid (1 g tissue with 7.5 mL). The homogenates were centrifuged at 10,000 × g for 20 min at 4°C twice, and the supernatants were gotten.

*(2) HPLC-FLD Analysis for DA and NE*. DA and NE in brain tissue of the rats were detected rapidly and simultaneously by HPLC-FLD [34]. The instrument parameters used for HPLC-FLD were as follows: Agilent chromatographic system; Agilent ZORBAX C18 column (4.6 mm × 250 mm, 5 *μ*m); mobile phase: methanol-buffer (10 : 90); the buffer consisted of 0.1 mol/L NaAc and 0.1 mmol/L EDTA-2Na (pH 5.1 with hydrochloric acid); flow rate: 1.0 mL/min; and column temperature: 30°C. Excitation and emission of the fluorescence detector were set to 280 and 330 nm, respectively. The area of peak amplitude was detected, and the levels of detecting index were calculated by a standard curve. The tissue levels of DA and NE were expressed in terms of nanograms per gram of tissue.

*(3) HPLC-UV Analysis for Alkaloids*. Agilent 1100 HPLC system consisting of a UV detector was used with Agilent ZORBAX C18 column (250 × 4.6 mm, 5 *μ*m) in 25°C column temperature. The components in brain tissue of the rats were detected simultaneously by HPLC-UV. The instrument parameters used for HPLC-UV were as follows: mobile phase: acetonitrile-buffer (50 : 50), the buffer consisted of 0.1 mol/L KH_2_PO_4_ and 0.015 mol/L sodium laurylsulfonate (pH 4.0 with phosphoric acid); and flow rate: 1.0 mL/min. An ultraviolet detector was set to 345 nm. The method for analysis and calculation of the components were referred to *Pharmacopoeia of the People's Republic of China*, which is a legal method for the quality control of TCM. Chromatographic peak of each alkaloid was identified by comparing their retention time against the known standard: berberine hydrochloride [35]. The area of peak amplitude was detected, and the levels of alkaloids were calculated. The tissue levels of components in the brains were expressed in terms of nanograms per gram of tissue.

### 2.6. Statistical Analysis

All data represented at least three independent experiments, and results of the experimental studies were expressed as mean ± standard deviation (SD). Statistical significance of differences was analysed by the Student *t*-test or one-way analysis of variance followed by the Bonferroni or Dunnett post hoc tests (GraphPad Prism Software, San Diego, CA, USA). *P* ≤ 0.05 were considered statistically significant.

## 3. Results

### 3.1. Network Pharmacological Analysis of HL-BX on DGP

#### 3.1.1. Potential Active Components of HL-BX

After searching the TCMSP database for the potential active ingredients of HL-BX, 116 compounds were found in BX and 48 compounds in HL. OB ≥ 30% and DL ≥ 0.18 were used as the screening conditions; 14 compounds in HL and 13 compounds in BX were found, as shown in Table 1.

#### 3.1.2. Compound-Target Network of HL-BX

**Figure 2:**
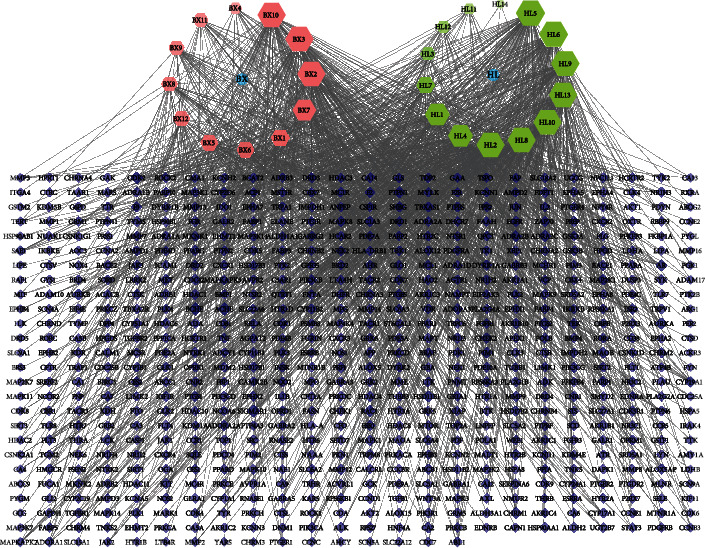
Compound-target network of HL-BX. The blue nodes represented TCM, the red nodes represented compounds in BX, the green nodes represented compounds in HL, and the purple nodes represented targets.

Compound-target network of HL-BX included 644 nodes (26 compound nodes, 616 target nodes) and 1663 edges. BX13 did not find the corresponding target in the database and did not participate in network construction. From the perspective of compounds, HL5 ((R)-canadine), HL6 (oxyberberine) and HL9 (palmatine) were the top three compounds, which could interact with 105, 103, and 102 target proteins, respectively. And from the perspective of target proteins, ACHE, BCHE, and CDK2 were the top three in the degree value, which could interact with 18, 17, and 14 compounds, respectively (Figure 2).

#### 3.1.3. Intersection Gene of HL-BX and DGP and Construction of PPI

**Figure 3:**
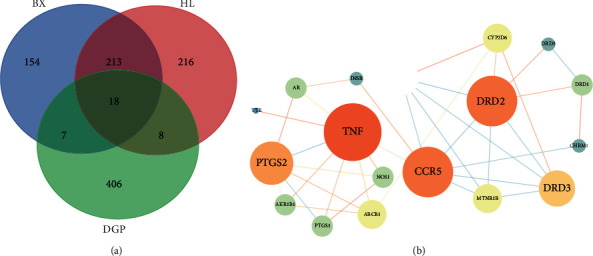
Venn diagram of gene intersection and PPI network of HL-BX and DGP: (a) Venn diagram of gene intersection between HL-BX and DGP; (b) PPI network of HL-BX and DGP.

Through the database, 392 possible active targets of compounds in BX and 455 possible active targets of compounds in HL were found, and 439 genes were related to DGP. Through the online Venn diagram, there were 213 genes in HL and BX, 8 genes in HL and DGP, 7 genes in BX and DGP, and 18 genes in HL-BX and DGP (Figure 3(a)). Then, the interaction of 18 target proteins was analysed by using the STRING database. The results were imported into the Cytoscape software for topological analysis. The results showed that the protein interactions involved 18 nodes and 36 edges (Figure 3(b)). Nodes represented proteins, and each edge represented the interaction between protein and protein. The larger the node, the larger the gray value, and the brighter the colour. The thicker the line, the darker the colour, representing the greater the correlation. The results showed that TNF, DRD2, and CCR5 may play a role in the treatment of DGP with HL-BX.

#### 3.1.4. GO Enrichment and KEGG Pathway Enrichment Analysis

**Figure 4:**
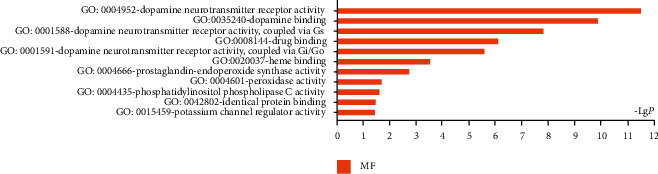
GO enrichment analysis of targets both in HL-BX and DGP.

18 interaction targets were imported into DAVID v6.8 for GO enrichment analysis. 11 GO terms (*P* < 0.05) were gotten in the enrichment of GO molecular function (MF). From the perspective of pathway, dopamine neurotransmitter receptor activity, dopamine binding, and dopamine neurotransmitter receptor activity (coupled *via* Gs) were the top three targets (Figure 4). According to the results, it was speculated that the efficacy of HL-BX on DGP might be mainly relevant to dopamine-related molecular functions by the crossgenes of HL-BX and DGP. KEGG pathway enrichment analysis was conducted. 18 targets and 6 pathways were screened (*P* < 0.05). The pathways were including neuroactive ligand-receptor interaction, dopaminergic synapse, regulation of lipolysis in adipocytes, calcium signalling pathway, cAMP signalling pathway, and serotonergic synapse. The details are shown in Table 2. It further indicated that HL-BX may achieve the treatment of DGP through multiple targets and pathways.

### 3.2. Evaluation of BX-HL Effects on DGP Rats *In Vivo*

In order to verify the results of network pharmacological analysis, further experiments *in vivo* were carried out. DGP rats were used to systematically evaluate the effects and the active components of HL-BX from the aspects of blood glucose, gastric emptying, intestinal propulsion, neurohormones, and neurotransmitters. According to clinical application and pharmacological and toxicological research progress of HL-BX [32, 36], three doses of 0.43 g/kg, 1.52 g/kg, and 4.3 g/kg were used, and no toxicity was observed.

#### 3.2.1. Effects of HL-BX on the Improvement of Fasting Blood Glucose, Gastric Emptying, and Intestinal Propulsion in DGP Rats

**Figure 5:**
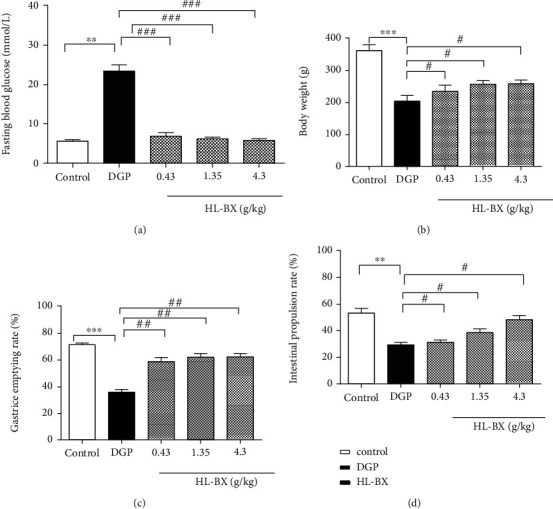
Effects of HL-BX on the improvement of fasting blood glucose, gastric emptying, and intestinal propulsion in DGP rats. DGP rats were treated with HL-BX (0.43, 1.35, and 4.3 g/kg) for 3 weeks. Fasting blood glucose was determined from the tail vein by using an Accu-Chek® Advantage blood glucose meter (a). The weight was determined (b). Gastric emptying rate (c) and intestinal propulsion rate (d) were calculated after 0.5 hour of intragastric administration with activated carbon.

DGP rats, induced by STZ and fed with a high-sucrose/high-fat diet for 4 weeks, received intragastric administration of HL-BX (0.43, 1.35, and 4.3 g/kg, respectively) for 3 weeks. Compared with the control group, the fasting blood glucose increased significantly (*P* < 0.001, Figure 5(a)) and the weights decreased significantly (*P* < 0.001, Figure 5(b)) in the model group. The gastric emptying rate and intestinal propulsion rate were both significantly reduced (*P* < 0.001, *P* < 0.01, respectively; Figures 5(c) and 5(d)) in the model group. These above indicated the success of the DGP model construction. Compared with the model group, the fasting blood glucose decreased significantly (*P* < 0.001) and the weights increased significantly (*P* < 0.05) in a dose-dependent manner with HL-BX (Figures 5(a) and 5(b)). The gastric emptying rates of HL-BX groups were 58.5 ± 3.2%, 61.9 ± 2.8%, and 62.2 ± 2.4% compared with 35.7 ± 2.1% in the model group, and the intestinal propulsion rates were 31.2 ± 1.7%, 38.5 ± 2.8%, and 48.2 ± 3.1% compared with 29.3 ± 1.9% in the model group (Figures 5(c) and 5(d)). The results showed that HL-BX could relieve the symptoms of DGP rats by reducing FBG and accelerating gastric emptying and intestinal propulsion.

#### 3.2.2. Effects of HL-BX on MLT, SP, VIP, SS, GLP-1, and 5-HT in the Serum of DGP Rats

**Figure 6:**
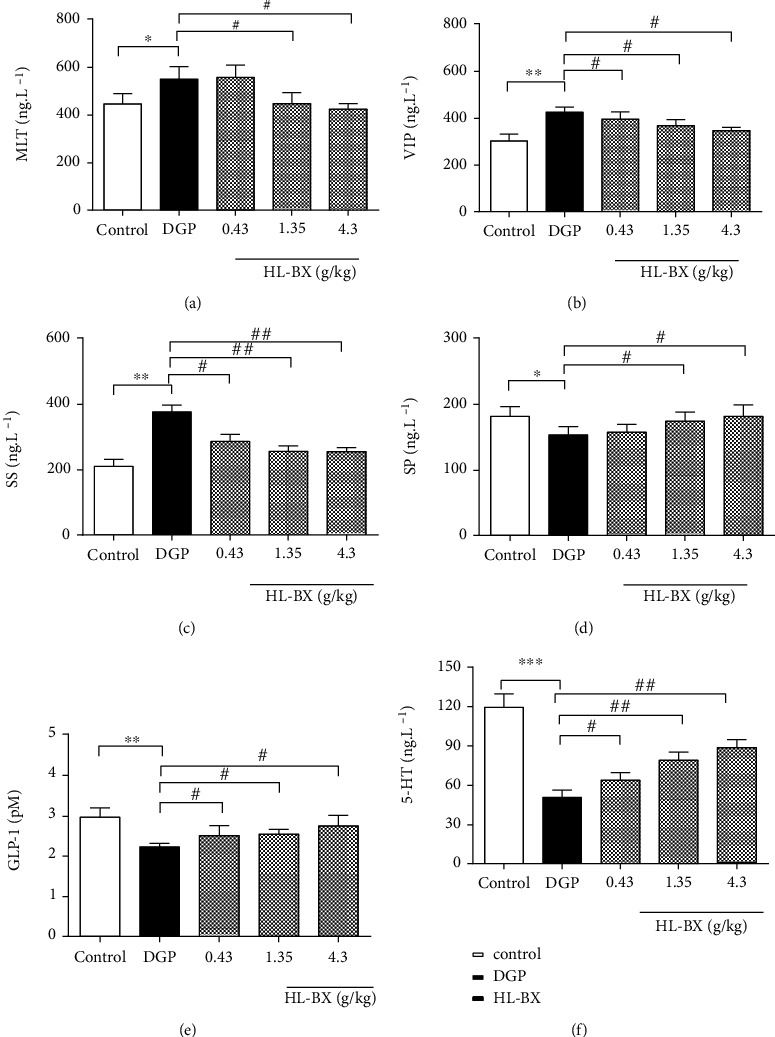
Effects of HL-BX on MLT, SP, VIP, SS, GLP-1, and 5-HT in the serum of DGP rats. After intragastric administration for 3 weeks, all rats were anesthetized and sacrificed. The serum were collected. The levels of MLT (a), SP (b), VIP (c), SS (d), GLP-1 (e), and 5-HT (f) in the serum were measured by ELISA.

Gastrointestinal motility was closely connected with a variety of neurohormones, such as MLT, SP, VIP, SS, GLP-1, and 5-HT, which regulated the balance between appetite and energy consumption through different metabolic pathways and neuronal pathways [37]. The hormonal dysfunction was one of the pathogeneses of delayed gastric emptying for DGP [3]. Based on the effects of HL-BX on the improvement in DGP rats, the levels of neurohormones (MLT, SP, VIP, SS, GLP-1, and 5-HT) were further investigated. Compared with the control group, MLT, VIP, and SS in the model group increased significantly (*P* < 0.05, *P* < 0.01, and *P* < 0.01, respectively; Figures 6(a)–6(c)), and the levels of SP, GLP-1, and 5-HT decreased significantly (*P* < 0.05, *P* < 0.01, and *P* < 0.001, respectively; Figures 6(d)–6(f)). Compared with the model group, the levels of MLT, VIP, and SS in the HL-BX treated groups decreased (*P* < 0.05, *P* < 0.05, and *P* < 0.01, respectively; Figures 6(a)–6(c)), and the levels of SP, GLP-1, and 5-HT of the HL-BX treated groups increased (*P* < 0.05, *P* < 0.05, and *P* < 0.01, respectively; Figures 6(d)–6(f)). These data showed that HL-BX could prevent and treat DGP by decreasing MLT, VIP, and SS and increasing SP, GLP-1, and 5-HT.

#### 3.2.3. Effects of HL-BX on DA and NE in Brain Tissue of DGP Rats

The brain is an important regulator of gastrointestinal movement and systemic glucose metabolism. As an important neurotransmitter, dopamine (DA) regulated not only brain functions but also a variety of behaviours including motor activity, food intake, and endocrine regulation [34]. These were all close to the improvement of gastrointestinal symptoms for DGP [38]. It was demonstrated that DA could affect whole-body glucose metabolism with increasing hepatic and peripheral insulin sensitivity and improving glucose tolerance in the series of human and rodent experiments [39]. Therefore, the effects of HL-BX on DGP might have something to do with the level of dopamine.

**Figure 7:**
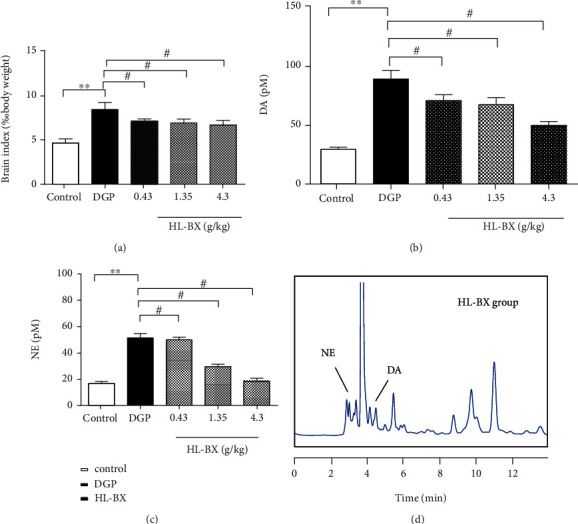
Effects of HL-BX on DA and NE in brain tissue of DGP rats. After intragastric administration for 3 weeks, all rats were anesthetized and sacrificed. Brain tissue was collected and homogenized. The supernatant was reserved by centrifugation with 6% perchloric acid, dried with nitrogen, redissolved with 200 *μ*L double steam water, and injected in HLPC. The brain index was calculated by brain tissue/the body weight (a). DA (b) and NE (c) were measured by HPLC. Chromatogram of HL-BX group by HPLC (d).

In this article, the levels of DA and its metabolite norepinephrine (NE) in the brain of the rats were detected. The method of simultaneous detection of DA and NE was used according to the previous literature [34]. The contents of DA and NE were calculated from their respective standard curves. Compared with the control group, the brain index of the model group increased significantly (*P* < 0.01, Figure 7(a)), and the levels of DA and NE in the model group increased significantly (*P* < 0.01, Figures 7(b) and 7(c)). Compared with the model group, the brain index of HL-BX-treated group decreased significantly (*P* < 0.01) and the levels of DA and NE in the HL-BX-treated groups declined (*P* < 0.05). These data showed that HL-BX could decrease DA and NE in brain tissue to prevent and treat DGP.

#### 3.2.4. Components Analysis of HL-BX in the Brain of DGP Rats

**Figure 8:**
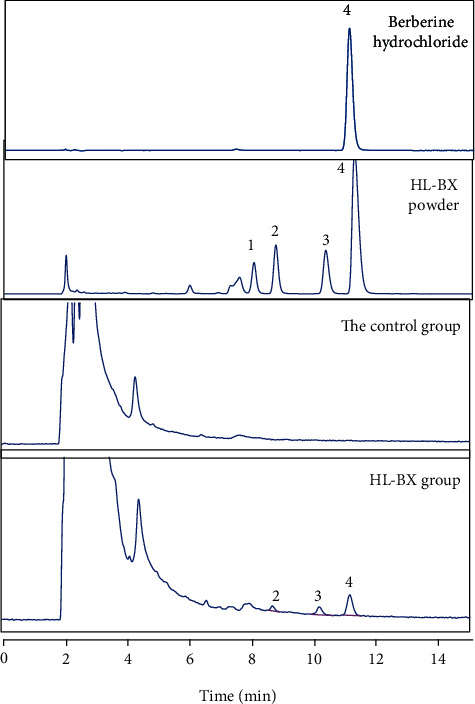
Chromatogram of components in by HPLC: (1) epiberberine; (2) coptisine; (3) palmatine; (4) berberine.

It was speculated that the effects of HL-BX on neurotransmitter DA and NE in brain tissue may be related to the absorption of components of HL-BX into the brain. Therefore, the components from HL-BX in the brain were analysed by HPLC. Epiberberine, coptisine, palmatine, and berberine were detected in HL-BX powder. None of the four substances were detected in the brain tissue of the model group rats or the 0.43 g/kg HL-BX group. There were three substances detected in the brain of 1.35 and 4.3 g/kg HL-BX groups (Figure 8). In brain tissue of the 1.35 g/kg HL-BX group, the contents of coptisine, palmatine, and berberine were 2.11 ± 0.13, 3.04 ± 0.15, and 9.35 ± 0.19 ng/g, respectively, while 7.45 ± 0.23, 8.05 ± 0.32, and 20.88 ± 0.21 ng/g in the brain tissue of the 4.3 g/kg group, respectively (Table 3). It suggested that three components including coptisine, palmatine, and berberine may play a vital role through the blood-brain barrier and they should be the most possibly biological components of HL-BX in the prevention and treatment of DGP.

## 4. Discussion

In this article, a practical application of TCM network pharmacological approach was used for the research on herb pair HL-BX in the treatment of DGP, and the results demonstrated that this approach was an effective strategy for TCM modern research. Firstly, twenty-seven potential active components of HL-BX were screened from the TCMSP database, and their potential targets were also retrieved. Then, the compound-target network and PPI network were constructed from predicted common targets, and several key targets were found based on the degree of the network. Next, GO and KEGG enrichment analyses were conducted to obtain several significantly enriched terms. Finally, the experimental verification was done. HL-BX had ameliorative effect on DGP rats by reducing FBG and accelerating gastric emptying and intestinal propulsion. HL-BX decreased the levels of MLT, VIP, and SS and increased the levels of SP, GLP-1, and 5-HT by reducing the levels of neurotransmitter DA and NE in the brain tissue of DGP rats. For the first time, three ingredients, coptisine, palmatine, and berberine from HL-BX, were traced in the brain tissue. It indicated that HL-BX regulated neurohormones by reducing neurotransmitters DA and NE through neuroactive ligand-receptor interaction to improve the symptoms of gastroparesis. The practical application of the network-based approach in HL-BX in the treatment of DGP demonstrated that this approach was an effective strategy and provides a scientific basis for the clinical application of HL-BX.

DGP is a serious and chronic complication of long-standing diabetes mellitus mainly characterized with delayed gastric emptying in the absence of mechanical obstruction. The coordinated digestive and reflexive processes in the upper gastrointestinal tract are under vagal modulatory control. The afferent vagus conveys visceral sensory information to the dorsal vagal complex, where it is integrated with information from other central nervous system centres involved in the regulation of autonomic and homeostatic functions [40]. Dopamine (DA), as an important neurotransmitter, played a significant role. It was reported that exogenously administered DA induced a profound gastroinhibition. And the inhibitory response was reduced by the DA2-like receptor antagonist [41]. Furthermore, it has been reported that gastric motility was modulated *via* activation of neuroactive ligand-receptor interaction, especially excitatory DA-like receptors on the A2 area [41]. Our results showed that the levels of neurotransmitter DA and its metabolite were increased in the brain tissue of DGP rats. This result was consistent with GO enrichment analysis of molecular function (MF), which showed dopamine neurotransmitter receptor activity, dopamine binding, and dopamine neurotransmitter receptor activity (coupled *via* Gs) were the top three targets (Figure 4). And this result was both consistent with KEGG enrichment analysis, which showed that neuroactive ligand-receptor interaction was the top pathway (Table 3). It is reasonable to conclude that neurotransmitter DA was the main factor of DGP and involved in the development and progression of DGP *via* neuroactive ligand-receptor interaction. Moreover, our results showed that HL-BX reduced the levels of neurotransmitter DA and its metabolite in brain tissue of DGP rats. It further suggested that HL-BX exerted an anti-DGP effect by reducing the levels of neurotransmitter DA and its metabolite *via* neuroactive ligand-receptor interaction.

Gastric emptying was the process of ejecting the stomach's content (chyme) into the duodenum. Several physiological factors, including fundal relaxation, pyloric control of flow into the duodenum, and antroduodenal coupling, affected the normal rate of gastric emptying [42]. As the high-activity hormones of the gut-brain axis, serum neurohormones were secreted by the gastrointestinal mucosa and pancreatic endocrine cells and regulated by the brain, which can stimulate or inhibit gastrointestinal motility [43]. The disordered neurohormones were principal features of the delayed gastric emptying [44, 45]. These neurohormones are released into the blood and act on target organs to exert regulatory effects, as well as affect adjacent cells through paracrine. In addition, some neurohormones act as neurotransmitters through nerve endings [44]. It was demonstrated that SS inhibited the secretion of digestive juices such as pancreatic exocrine and bile secretion, weakened contraction of gastrointestinal smooth muscle, and delayed gastrointestinal emptying. VIP inhibited gastrointestinal motility by impairing intestinal contraction and spontaneous pyloric contraction [46]. MLT was produced in the small intestine. During the fasting state, plasma levels of MLT fluctuated and induced gastric contractions to signal hunger *via* a cholinergic pathway. Targeting the MLT receptor has therapeutic potential to treat hypomotility disorders, modulate hunger, and affect glucose metabolism [47]. The concentrations of GLP-1 [48] and 5-HT [49] were lower in diabetes patients with gastroparesis, and exogenous provision of 5-HT reversed delayed gastric emptying. Smiley, Smiley et al. reported that the level of SP was significantly decreased in DGP rats [50]. In this study, MLT, VIP, and SS were significantly increased in DGP model rats, while the concentrations of SP, GLP-1, and 5-HT were significantly decreased. HL-BX restored the levels of neurohormone to varying degrees. It indicated that neurohormones MLT, VIP, SS, SP, GLP-1, and 5-HT regulated by the neurotransmitters though the gut-brain axis were involved in the development and progression of DGP. These were consistent with the above reports. A recent systematic review of randomized controlled trials showed that Ban-Xia-Xie-Xin decoction could regain the gastric emptying rate and improve diabetic gastrointestinal symptoms of diabetes mellitus [7]. As the core pair of Ban-Xia-Xie-Xin decoction, HL-BX improved gastric emptying and intestinal propulsion by increasing the levels of SP, GLP-1, and 5-HT and decreasing MLT, VIP, and SS in our study. It further indicated that HL-BX exerted an anti-DGP effect to regulate neurohormones by the neurotransmitters of the gut-brain axis.

In the past decades, herb pair has been highly valued as the smallest compatibility units in TCM formula [51]. HL-BX became a classic herb pair in the treatment of DGP. In the current study, we discovered that twenty-seven compounds were potential active components of HL-BX based on network pharmacology. There were three of them confirmed in brain tissue by HPLC. They were coptisine, palmatine, and berberine, corresponding to HL11, HL1, and HL9, respectively, by network pharmacological analysis (Table 1). Further verification is needed to do with the others predicted. On the other hand, it was documented that the inhibitory effects of HL and berberine on the repeated cocaine-induced locomotor activity were close to the decrease of dopamine biosynthesis and postsynaptic neuron activity [52]. Berberine lowered triglyceride by the upregulation of lipolysis gene expressions and downregulation of lipogenesis gene expressions through the activation of the AMPK signalling pathway [53]. Palmatine had a neuroprotective effect *via* inhibiting oxidative stress and inflammation in the dopaminergic system [54]. These were consistent with the signalling pathway predicted by network pharmacology to alleviate DGP. Our results showed that neurotransmitter DA was the main factor of DGP and involved in the development and progression of DGP *via* neuroactive ligand-receptor interaction. However, other pathways have not been confirmed. These screened targets and pathways resulted from network pharmacological analysis provided the future research direction for the mechanism study of drug pair HL-BX. In general, these findings indicated that network pharmacology could advance the comprehensive understanding of TCM.

Network pharmacology, integrated systems biology, bioinformatics, network science, and other disciplines have become a useful tool in identifying alternative targets for herbal medicines and mechanisms of action for TCM. It focuses on the complex interactions in biological systems from an overall perspective of the system level and biological network, rather than altering the single molecular component. Although our results have demonstrated that this approach was beneficial for TCM modern research, the application of TCM network pharmacological approach was procedurally and simply used in current researches. Recently, the network pharmacology evaluation method guidance was issued [55]. It required network pharmacology evaluation about data collection, network analysis, and result verification from three aspects of reliability, standardization, and rationality. Compared with the guidance, there are still some deficiencies in this paper and our research is still in its infancy. Due to the limitations of current research level and conditions, there are still many problems to be solved.

1. Database and threshold value. Only one database (TCMSP) was used to collect the chemical components of HL-BX, and only Swiss Target Prediction was used to predict the targets without comprehensive analysis of multiple databases. And a single parameter was occasionally screened; for instance, minimum required interaction score was used when using STRING in the construction of PPI network. As a result, the richness and accuracy of data information may be insufficient as a consequence of the lack of reliability and rationality. There are various databases and data resources related to TCM, such as TCM-ID (Traditional Chinese Medicine Information Database), TCM Database@Taiwan, and TCMID (Traditional Chinese Medicine Integrated Database). Further, using comprehensive databases and selecting reasonable threshold value would enrich our research in the future.
2. Result verification. There were 27 potential active ingredients of HL-BX screened from the database, but only 3 ingredients were verified. The potential role of other compounds should not be ignored. The others remain to be confirmed. In order to identify those compositions, the detection method with high sensitivity, such as HPLC-MS, might be used. Considering the metabolic process *in vivo*, the components and their metabolites may be both detected. At the same time, experimental verification *in vivo* and in *vitro* should both be carried out. 18 targets and 6 pathways were retrieved from GO enrichment and KEGG pathway enrichment analysis, and only neuroactive ligand-receptor interaction was analysed in the current experiment. Target and pathway verification should be gradually supplemented by the scientific method, such as Western blot, PCR, and molecular docking, to provide an experimental basis for systematically and comprehensively explaining the effect of HL-BX on DGP in the future.

## 5. Conclusions

To date, a practical application of TCM network pharmacological approach was used for the research on herb pair HL-BX. We present the first evidence of the effects, compounds, and pathways of herb pair HL-BX on DGP. The anti-DGP activities of the HL-BX were identified in this work through network pharmacology methods and can serve as potential anti-DGP ingredients for future experimental validation. The results demonstrated that this approach was an effective strategy for TCM modern research. The application of network pharmacological approach could advance the comprehensive understanding of TCM and promote the modernization of TCM.

## Figures and Tables

**Table 1:** Potential active components of HL-BX.

ID	Mol ID	Molecule name	OB%	DL	MW	Herb name
HL1	MOL001454	Berberine	36.86	0.78	336.39	Huanglian
HL2	MOL013352	Obacunone	43.29	0.77	454.56	Huanglian
HL3	MOL002894	Berberrubine	35.74	0.73	322.36	Huanglian
HL4	MOL002897	Epiberberine	43.09	0.78	336.39	Huanglian
HL5	MOL002903	(R)-Canadine	55.37	0.77	339.42	Huanglian
HL6	MOL002904	Oxyberberine	36.68	0.82	351.38	Huanglian
HL7	MOL000622	Magnograndiolide	63.71	0.19	266.37	Huanglian
HL8	MOL000762	Palmidin A	35.36	0.65	510.52	Huanglian
HL9	MOL000785	Palmatine	64.60	0.65	352.44	Huanglian
HL10	MOL000098	Quercetin	46.43	0.28	302.25	Huanglian
HL11	MOL001458	Coptisine	30.67	0.86	320.34	Huanglian
HL12	MOL002668	Worenine	45.83	0.87	334.37	Huanglian
HL13	MOL008647	Moupinamide	86.71	0.26	313.38	Huanglian
HL14	MOL002907	Corchoroside A_qt	104.95	0.78	404.55	Huanglian
BX1	MOL001755	Stigmast-4-en-3-one	36.08	0.76	412.77	Banxia
BX2	MOL002670	Cavidine	35.64	0.81	353.45	Banxia
BX3	MOL002714	Baicalein	33.52	0.21	270.25	Banxia
BX4	MOL002776	Baicalin	40.12	0.75	446.39	Banxia
BX5	MOL000358	Beta-sitosterol	36.91	0.75	414.79	Banxia
BX6	MOL000449	Stigmasterol	43.83	0.76	412.77	Banxia
BX7	MOL005030	11-Eicosenoic acid	30.70	0.20	310.58	Banxia
BX8	MOL000519	(+)-Neocryptotanshinone	31.11	0.32	314.41	Banxia
BX9	MOL006936	Methyl eicosa-10,13-dienoate	39.99	0.20	308.56	Banxia
BX10	MOL006957	Cyclo(L-tyrosyl-L-phenylalanyl)	46.89	0.27	310.38	Banxia
BX11	MOL003578	Cycloartenol	38.69	0.78	426.80	Banxia
BX12	MOL006967	Xanthosine	44.72	0.21	284.26	Banxia
BX13	MOL006937	12,13-Epoxy-9-hydroxynonadeca-7,10-dienoic acid	42.15	0.24	324.51	Banxia

**Table 2:** Information of potential targets and signalling pathways for constituents from HL-BX.

No.	Pathway name	Genes	Count	Hits	*P*
1	Neuroactive ligand-receptor interaction	DRD1, DRD3, DRD2, DRD5, CHRM1, DRD4, MTNR1B	7	277	2.30*E*-05
2	Dopaminergic synapse	DRD1, DRD3, DRD2, DRD5, DRD4	5	128	1.75*E*-04
3	Regulation of lipolysis in adipocytes	PTGS2, PTGS1, INSR	3	56	0.00726
4	Calcium signalling pathway	DRD1, NOS1, DRD5, CHRM1	4	179	0.007558
5	cAMP signalling pathway	DRD1, DRD2, DRD5, CHRM1	4	198	0.009973
6	Serotonergic synapse	PTGS2, PTGS1, CYP2D6	3	111	0.026721

**Table 3:** The contents of components detected in brain tissue (ng/g).

Group	Coptisine (ng/g)	Palmatine (ng/g)	Berberine (ng/g)
1.35 g/kg HL-BX group	2.11 ± 0.13	3.04 ± 0.15	9.35 ± 0.19
4.3 g/kg HL-BX group	7.45 ± 0.23	8.05 ± 0.32	20.88 ± 0.21

## Data Availability

The data used to support the findings of this study are included within the article.
